# CRISPR-Cas system positively regulates virulence of *Salmonella enterica* serovar Typhimurium

**DOI:** 10.1186/s13099-024-00653-5

**Published:** 2024-10-26

**Authors:** Nandita Sharma, Ankita Das, Abhilash Vijay Nair, Palash Sethi, Vidya Devi Negi, Dipshikha Chakravortty, Sandhya Amol Marathe

**Affiliations:** 1 Department of Biological Sciences, Birla Institute of Technology & Science, Pilani, Rajasthan 333031 India; 2 Department of Microbiology and Cell Biology, Indian Institute of Science, Bangalore, Karnataka 560012 India; 3 Department of Biological Sciences, Indian Institute of Science Education and Research Mohali, Punjab, 140306 India

**Keywords:** *Salmonella*, Type 1-E CRISPR-Cas system, Virulence, SPI-1-T3SS, SPI-2-T3SS, Anti-oxidant genes

## Abstract

**Background:**

*Salmonella*, a foodborne pathogen, possesses a type I-E clustered regularly interspaced short palindromic repeats (CRISPR)-CRISPR associated (Cas) system. We investigated the system’s role in regulating *Salmonella* virulence by deleting the CRISPR arrays and Cas operon.

**Results:**

Our study demonstrates invasion and proliferation defects of CRISPR-Cas knockout strains in intestinal epithelial cells and macrophages owing to the repression of invasion and virulence genes. However, proliferation defects were not observed in the Gp91^phox−/−^ macrophages, suggesting the system’s role in the pathogens’ antioxidant defense. We deduced that the CRISPR-Cas system positively regulates H_2_O_2_ importer (OmpW), catalase (*katG*), peroxidase (*ahpC*), and superoxide dismutase (*soda* and *sodCI*), thereby protecting the cells from oxidative radicals. The knockout strains were attenuated in in-vivo infection models (*Caenorhabditis elegans* and BALB/c mice) due to hypersensitivity against antimicrobial peptides, complement proteins, and oxidative stress. The attenuation in virulence was attributed to the suppression of LPS modifying (*pmr*) genes, antioxidant genes, master regulators, and effectors of the SPI-1 (invasion) and SPI-2 (proliferation) islands in knockout strains. The regulation could be attributed to the partial complementarity of the CRISPR spacers with these genes.

**Conclusions:**

Overall, our study extends our understanding of the role of the CRISPR-Cas system in *Salmonella* pathogenesis and its virulence determinants.

**Supplementary Information:**

The online version contains supplementary material available at 10.1186/s13099-024-00653-5.

## Introduction

The bacterial adaptive immune system, Clustered Regularly Interspaced Short Palindromic Repeats (CRISPR) and CRISPR-associated (Cas) endonucleases, acts against invading mobile genetic elements. Besides their canonical functions, the CRISPR loci and Cas proteins may independently regulate host genes involved in the physiology and virulence of various bacteria [1], including *Streptococcus* [2], *Enterobacter* [3], *Francisella* [4], *Campylobacter* [5], *Neisseria* [6], *Listeria* [7], *Pseudomonas* [8] and *Salmonella* [9]. However, the mechanistic details have not yet been investigated thoroughly.

Shariat et al. traced 15% of the *Salmonella* proto-spacers on the chromosome instead of its general targets, phages, and plasmids, but they did not identify the target genes [10]. Recent studies have started to uncover the significant role of the CRISPR-Cas system in regulating *Salmonella* physiology and virulence. For instance, the expression of *cas7* was detected in human macrophages infected by *S*. Typhi. The transcriptome profile of bacteria displayed altered expression of *cas* genes in clinical *S.* Typhi samples, suggesting the role of the type I-E CRISPR-Cas system during *Salmonella* infection [11]. A similar transcriptomic study by Eriksson et al. for intra-macrophage *Salmonella enterica* subspecies *enterica* Typhimurium (*S*. Typhimurium) detected a change in the expression of *cas3* gene [12]. A recent study by Cui et al. on *Salmonella enterica* subspecies *enterica* Enteritidis (*S*. Enteritidis) correlates the importance of the system in regulating quorum sensing, biofilm formation, and bacterial invasion into the host [9]. Another relevant study was performed by Stringer et al. on *S*. Typhimurium, where ChIP seq analysis confirmed 236 crRNA and Cascade-binding sites on the bacterial genome [13]. Reportedly, some of these Cascade-binding sites are within the virulence genes [14], implying the regulation of pathogenic traits by the CRISPR-Cas system. Furthermore, the system regulates the expression of a diverse array of genes associated with biofilm formation in *Salmonella* [15]. All these studies suggest remodeling of *Salmonella* pathogenicity by endogenous CRISPR-Cas systems. This conception can strongly influence the design of effective therapeutic strategies against salmonellosis, which presents a formidable threat to humans, causing typhoid fever in 14.3 million individuals, with 135,000 estimated deaths worldwide (World Health Organization (WHO), 2018) [16].

Building upon these findings, we hypothesized that the type I-E CRISPR-Cas system could modulate key virulence factors and host-pathogen interactions during *Salmonella* infection, affecting bacterial survival at various stages of the infection cycle. We found that the system regulates the virulence of *S*. Typhimurium, helping it evade host defenses by regulating important virulence genes.

## Materials and methods

### Bacterial strains, nematode, and culture conditions

This study used the wildtype *Salmonella enterica* serovar Typhimurium strain 14028s as the parent strain. We cultured the wildtype, knockout (Δ*crisprI*, Δ*crisprII*, ΔΔ*crisprI crisprII* and Δ*cas op)* and complement (Δ*crisprI + pcrisprI* and Δ*crisprII + pcrisprII*) strains in Luria-Bertani (LB, Himedia) with appropriate antibiotics (Supplementary Table 1) [15]. *Escherichia coli* OP50, the wildtype and knockout strains, were electro-transformed with pFPV-mCherry plasmid to obtain mCherry fluorescent derivatives. A wildtype N2 strain of *Caenorhabditis elegans* was routinely maintained at 25 °C on a nematode growth medium (NGM) agar plate with *E. coli* OP50 as a food source.

### Eukaryotic cell lines and growth conditions

The RAW 264.7 and HT-29 cell lines (obtained from NCCS, Pune) were grown in Dulbecco′s modified minimum essential medium (Gibco) and Roswell Park Memorial Institute 1640 media (Sigma Aldrich), respectively, with 10% fetal bovine serum (FBS, Himedia) at 37 °C temperature in the presence of 5% CO_2_. RAW 264.7 cells were activated with 10 ng/mL Lipopolysaccharide (LPS) from *E. coli* (Sigma) for 24 h. RPMI was supplemented with 2 mM glutaMAX™ (Gibco) for 15 days to polarize the HT-29 cells. Murine peritoneal macrophages were harvested from BALB/c, C57BL/6, and gp91^phox −/−^ mice, as described previously [17].

### Percentage phagocytosis/invasion assay

Bacterial phagocytosis and invasion were estimated using a gentamicin protection assay in macrophages and intestinal epithelial cell lines, respectively. RAW 264.7 and peritoneal macrophages were infected with stationary phase cultures of wildtype, Δ*crisprI*, Δ*crisprII*, Δ*cas op*, and ΔΔ*crisprI crisprII* knockout strains and their respective complement strains Δ*crisprI +* p*crisprI* and Δ*crisprII +* p*crisprII* at a multiplicity of infection (MOI) 5. MOI 10 was used to infect HT-29 cells. The cells were washed thrice with phosphate-buffered saline (PBS) and subjected to 100 µg/mL of gentamicin treatment for 1 h. The cells were rewashed with PBS and lysed with 0.5 mL of 0.1% Triton X-100 (Sigma). The colony forming units (CFU) were determined by serially diluting the lysates and plating them onto LB agar supplemented with appropriate antibiotics. Percentage phagocytosis/invasion was determined using the following formula:

$$\% \;invasion{\rm{ }}/phagocytosis\; = \;\frac{{CFU\;at\;1\;h}}{{CFU\;of\;pre - inoculum}}\; \times \;100$$

### Intracellular proliferation assay

We infected the macrophage and intestinal cell lines as mentioned above in the phagocytosis/invasion assay and lysed them at 2 h and 16 h post-infection. The serially diluted lysates were plated onto LB agar supplemented with antibiotics to determine Colony Forming Units (CFU) at 2 h and 16 h. The fold proliferation 16 h to 2 h was determined using the following formula:

$$Fold\;proliferation\; = \;\frac{{CFU\;at\;16h}}{{CFU\;at\;2h}}$$

#### In-vivo infection assay

For infection studies, we used 6–8 weeks BALB/c mice weighing 20–22 g raised in Central Animal Facility, Indian Institute of Science (IISc), Bangalore, as per the guidelines of the Institutional Animal Ethics Committee at the IISc, Bangalore, India. Five mice in five sets were orally gavaged with 10^7^ bacterial cells of wild type, Δc*risprI*, Δ*crisprII*, Δ*cas op*, and ΔΔ*crisprI crisprII* knockout strains. After 3 days post-infection, reticuloendothelial organs like the spleen, liver, Peyer’s patch (PP), and mesenteric lymph nodes (MLN) were aseptically isolated, weighed and homogenized in 0.5 mL of sterile PBS using a bead-beater (Bio spec products, USA). To obtain CFU per gram weight for each organ, serial dilutions of the homogenate were plated onto *Salmonella Shigella* agar (SS agar, Himedia) containing appropriate antibiotics.

#### Cytokine analysis

The blood from control and infected mice was collected on the 4th day by the retro-orbital bleeding method, and the sera were separated once the blood was clotted. The concentration of IFN-γ, IL-4, and IL-10 in the pooled sera of each set was estimated using a Thermo Fisher Scientific kit as per the manufacturer’s instructions.

### Bacterial colonization assay in *C. elegans*

The mCherry-tagged bacterial strains were grown overnight in LB broth at 37 °C, and lawns were prepared by spreading 200 µL of overnight bacterial culture on modified NGM agar. To measure the intestinal colonization of the test strains in *C. elegans*, the synchronized L4 larvae were exposed to fluorescently-tagged (m-Cherry) strains of wildtype, Δ*crisprI*, Δc*risprII*, Δ*cas op*, ΔΔ*crisprI crisprII*, and *E. coli* OP50. After 24 h, the worms were anesthetized with 25 mM levamisole (Sigma), washed thrice with M9 buffer, and treated with 80 µg/mL of gentamicin for 1 h, followed by treatment with 25 µg/mL of gentamicin for 30 min. Finally, the worms were washed with M9 buffer and lysed with 0.2% Triton X-100 (Sigma) in a tissue lyser LT (Qiagen, India). The lysates were serially diluted and plated on LB-agar containing ampicillin to estimate bacterial burden.

### Antimicrobial peptide killing assay

Overnight-grown bacterial cultures were subcultured at a ratio of 1:40 in Luria broth and incubated at 37 °C until the OD_600nm_ reached 0.3–0.4. 10^5^ bacterial cells were treated with polymyxin B (0.5 µg/mL, Himedia) and protamine sulfate (0.5 µg/mL, PROTA) in TN (0.5% tryptone and 0.5% NaCl) media for 1 h at 37 °C with slight agitation. Following the incubation, the mixture was plated onto LB-agar plates supplemented with appropriate antibiotics. Percentage survival was calculated with respect to the untreated samples.

### Serum sensitivity

Bacterial strains were grown overnight in Luria broth, and 10^7^ bacterial cells from an overnight culture were incubated in 20% FBS (Himedia) for 2 h at 37 °C with slight agitation. Serial dilutions of these cultures were plated onto LB agar supplemented with antibiotics to determine the CFU. The percentage survival was calculated using the following formula:

$$\% \;survival\; = \;\frac{{CFU\;of\;serum\;treated\;samples}}{{CFU\;of\;untreated\;samples}}\; \times \;100$$

### Measurement of intracellular reactive oxygen species (ROS)

LPS-activated RAW 264.7 cells were infected with wildtype, Δ*crisprI*, Δ*crisprII*, Δc*as op*,* and* ΔΔ*crisprI crisprII* knockout strains at MOI 5 as described in the above sections. The intracellular ROS was determined using an oxidant-sensitive probe 2′,7′- dichlorodihydrofluorescein diacetate (H_2_DCFDA, Sigma at 5 µM concentration) 6 h post-infection. The cells were washed with sterile PBS, and fluorescence intensity was measured at (λ_ex_) of 485 nm and (λ_em_) at 535 nm using Fluoroskan (Thermo Scientific).

### Measurement of extracellular reactive nitrogen species (RNS)

We measured extracellular nitrite as described previously [18]. RAW 264.7 cells were infected, as described in the above section. 50 µL the extracellular media were collected from cells infected with wildtype, Δ*crisprI*, Δ*crisprII*, Δ*cas op*,* and* ΔΔ*crisprI crisprII* knockout strains at 16 h post-infection and subjected to nitrite estimation by Griess reagents.

### Bacterial sensitivity to hydrogen peroxide

Overnight grown bacterial cultures of wildtype, Δ*crisprI*, Δ*crisprII*, Δ*cas op*, ΔΔ*crisprI crisprII*, Δ*crisprI +* p*crisprI* and Δ*crisprII +* p*crisprII* strains were treated with 1 mM H_2_O_2_ in Muller Hinton (MH, Himedia, pH- 5.4) media for 2 h. We plated the bacterial suspensions onto LB-agar plates containing appropriate antibiotics to determine Colony Forming Units (CFU).

#### Priming assay with hydrogen peroxide

The overnight grown bacterial cultures were subcultured in MH Media and grown to OD_600nm_ ∼ 0.4. For one set, we exposed the bacteria to 0.1 mM H_2_O_2_ (priming) for 30 min in the dark at 37 °C under shaking. The H_2_O_2_ was removed by centrifugation at 5000 x g for 10 min, and cells were allowed to recover for 2 h. The other set was left untreated. We subcultured equal amounts (10^7^) of bacteria from each set in MH media The bacteria were incubated with 0 mM and 1 mM (trigger) of H_2_O_2_ at 37 °C in the dark at 100 rpm. After 8 h, we determined the bacterial growth by measuring the OD_600nm_ using Multiskan GO (Thermo Scientific, USA).

The percentage survival was calculated using the following formula:

$$\% \;survival\; = \;\frac{{CFU/OD\;of\;{H_2}\;{O_2}\;treated\;samples}}{{CFU/OD\;of\;untreated\;samples}}\; \times \;100$$

### RNA isolation and quantitative real-time (q-RT) PCR

Bacterial strains grown overnight were subcultured at a ratio of 1:100 in LB (SPI-I inducing condition) and magnesium minimal medium MES (MgM-MES) (pH = 5.4, SPI-2 inducing condition) [19, 20]. The bacterial cells were incubated at 37 °C and 150 rpm for 8 h and 4 h, respectively. At the end of the specified incubation, the RNA was isolated using TRIzol reagent (Himedia), and cDNA was synthesized using iScript™ cDNA synthesis kit (Biorad). qRT-PCR was performed using PowerUp™ SYBR™ Green Master Mix (Thermo Fisher Scientific). Relative expression of the gene was calculated using the 2^−ΔΔCt^ method by normalizing to reference gene *rpoD*. The primers used in RT-qPCR are listed in (Supplementary Table 2).

#### In-silico analysis

We aligned the spacer sequences in CRISPR-I and CRISPR-II arrays with the coding and reverse complement sequences of different SPI-1 and SPI-2 genes using Serial Cloner version 2.6 software. Additionally, we screened for the presence of known protospacer adjacent motif (PAM) sequences in the vicinity.

### Statistical analysis

We performed statistical analysis using Prism 8 software (GraphPad, California). We present data as the mean ± standard deviation (S.D) and assess it through one-way ANOVA (Dunnett’s multiple comparison test) in at least three independent experiments with at least three technical replicates. The animal experiments are in two biological replicates with five technical replicates. Statistical significance is as follows: *p ≤ 0.05; ** *p* ≤ 0.01; *** *p* ≤ 0.001; **** *p* < 0.0001; and ns, not significant.

## Results

### Deletion of the CRISPR-Cas components reduced invasion and intracellular survival of the CRISPR-Cas knockout strains in cell culture infection models

To understand the role of the CRISPR-Cas system in the pathogenicity of *S*. Typhimurium, we assessed the ability of the wildtype and CRISPR-Cas knockout strains to invade and proliferate in intestinal epithelial cells and macrophages.

**Fig. 1:**
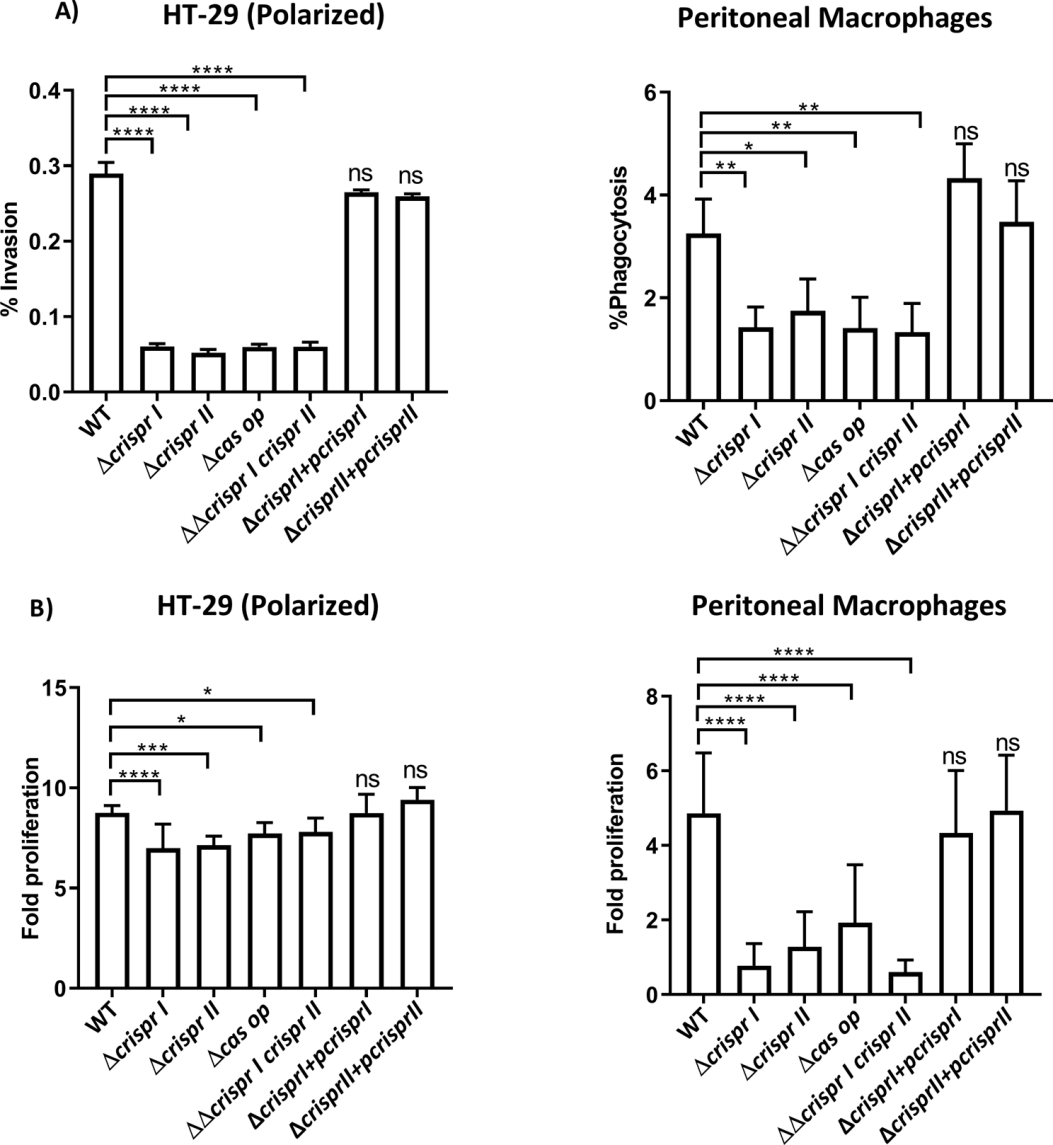
The knockout strains of CRISPR-Cas components show invasion and replication defects. **(A-B)** HT-29 cell lines, and peritoneal macrophages were infected with *S.* Typhimurium strain 14028s wildtype (WT), CRISPR (Δ*crisprI*, Δ*crisprII*, and ΔΔ*crisprI crisprII*) and *cas* operon *  (*Δ*cas op*) knockout strains along with their respective complements (Δ*crisprI +* p*crisprI* and Δ*crisprII +* p*crisprII*). **(A)** The percentage of invasion/ phagocytosis in intestinal epithelial cells was calculated using CFU analysis of the infected cell lysate and the pre-inocula used for infection. Fold proliferation was calculated by normalizing the CFU at 16 h to 2 h. One-way ANOVA (Dunnett’s multiple comparison test) was used to determine significant differences between the WT and knockout strains, in at least three independent experiments, with at least 3 replicates in each. Error bars indicate SD. Statistical significance is shown as follows: *, *p* ≤ 0.05; **, *p* ≤ 0.01; ***, *p* ≤ 0.001; ****, *p* < 0.0001; and ns, not significant

We evaluated the invasion and intracellular survival of the strains in non-polarized and polarized HT-29 cells. Compared to the wildtype strain, the knockout strains showed impaired invasiveness in the HT-29 cells, and the reintroduction of the knocked-out gene rescued the invasiveness. However, the CRISPR-Cas knockout strains exhibited an enhanced attenuation in percentage invasion in polarized cells (Fig. 1A and Supplementary Fig. S1A). Though the fold replication of all the strains was less in the polarized cells, the knockout strains showed reduced replication than that of the wildtype in both the cell types (Fig. 1A and Supplementary Fig. S1A). The reduction in fold replication of the knockout strains was 1.5-2 times in non-polarized HT-29, while in polarized cells, the fold replication of knockout strains was ∼ 1.3 times less than that of the wildtype strain (Fig. 1B and Supplementary Fig. S1B).

As illustrated in Fig. 1A and S1A, the CRISPR-Cas knockout strains exhibited a 35–60% reduction in phagocytosis by macrophages, compared to wildtype (Fig. 1A and Supplementary Fig. S1A). Moreover, the intracellular proliferation of the CRISPR-Cas knockout strains decreased by 1.5-2.5-fold in RAW 264.7 cell lines and by 2.5-5-fold in peritoneal macrophages (Fig. 1B and Supplementary Fig. S1B). In all these infection experiments, the complementation of corresponding genes in Δ*crisprI* and Δ*crisprII* showed a reversal of the phenotypes close to that of the wildtype, confirming that the gene deletion process is clean without any side/off-target effects. Thus, we did not use the complementary strains for future experiments.

### Deletion of the CRISPR-Cas components reduced the virulence of *Salmonella* Typhimurium in *in-vivo* infection models

**Fig. 2:**
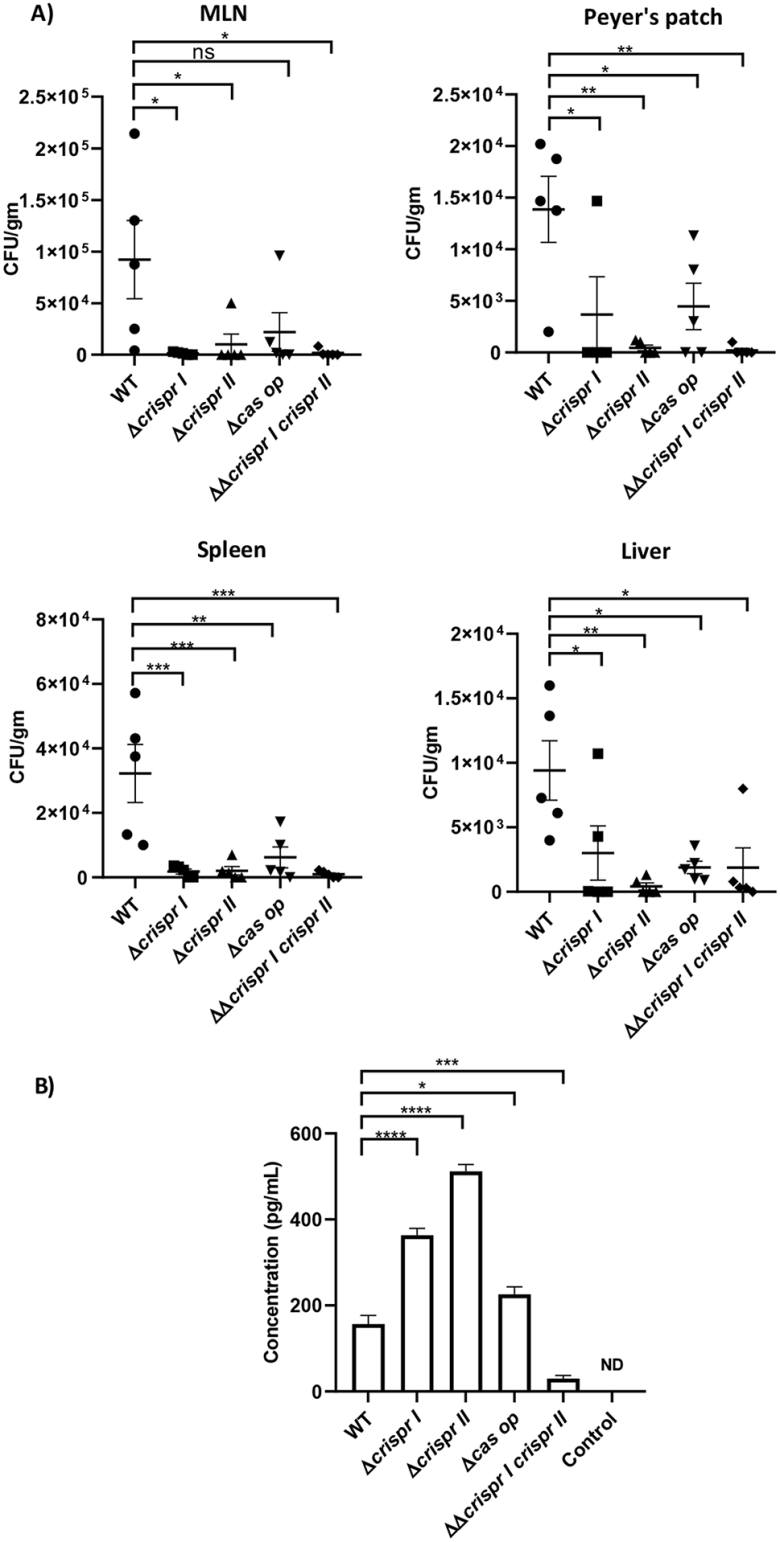
The knockout strains of CRISPR-Cas components show impaired colonisation in in-vivo model organism (mice) **(A)** The mice were orally gavaged with wildtype (WT) and CRISPR (Δ*crisprI*, Δ*crisprII*, and ΔΔ*crisprI crisprII)* and *cas* operon* (*Δ*cas op*) knockout strains. Bacterial burden in different reticuloendothelial organs of these infected mice was estimated 3 days post-infection by plating the organ lysates, followed by CFU analysis. **(B)** The sera of infected mice were pooled and the concentrations of proinflammatory cytokine IFN-γ was determined using ELISA. Results are represented as mean ± SD pooled sera samples (2 mice per pool) for each infected and control group. One-way ANOVA (Dunnett’s multiple comparison test) was used to determine significant differences between the WT and knockout strains, in two independent experiments, with at least 3 replicates in each. Error bars indicate SD. Statistical significance is shown as follows: *, *p* ≤ 0.05; **, *p* ≤ 0.01; ***, *p* ≤ 0.001; ****, *p* < 0.0001; and ns, not significant

Next, we tested the pathogenic potential of the knockout strains in *C. elegans* using the bacterial colonization assay. We observed a 40–60% reduction in the colonization of nematodes exposed to the CRISPR-Cas knockout strains compared to those exposed to the wildtype strain (Supplementary Fig. S2). These observations were further validated in the murine model of typhoid fever using BALB/c mice. We dissected the infected mice three days post-infection to enumerate the bacterial burden in the Peyer’s patch (PP), mesenteric lymph node (MLN), liver, and spleen. The knockout strains displayed significantly reduced bacterial load in all these organs (Fig. 2A), indicating the role of the CRISPR-Cas system in establishing in-vivo infection of *Salmonella.*

Further, we assessed the induction of inflammatory immune responses in the infected BALB/c mice. In serum, levels of the pro-inflammatory cytokine IFN-γ were elevated by approximately 2-2.5 fold compared to control mice infected with the wildtype strain (*p* < 0.0001), except in the case of the ΔΔ*crisprI crisprII* strain. The anti-inflammatory cytokines IL-4 and IL-10 were not detectable in the serum of the infected mice.

### The CRISPR array and *cas* operon knockout strains are susceptible to antimicrobial peptides, and the complement system

**Fig. 3:**
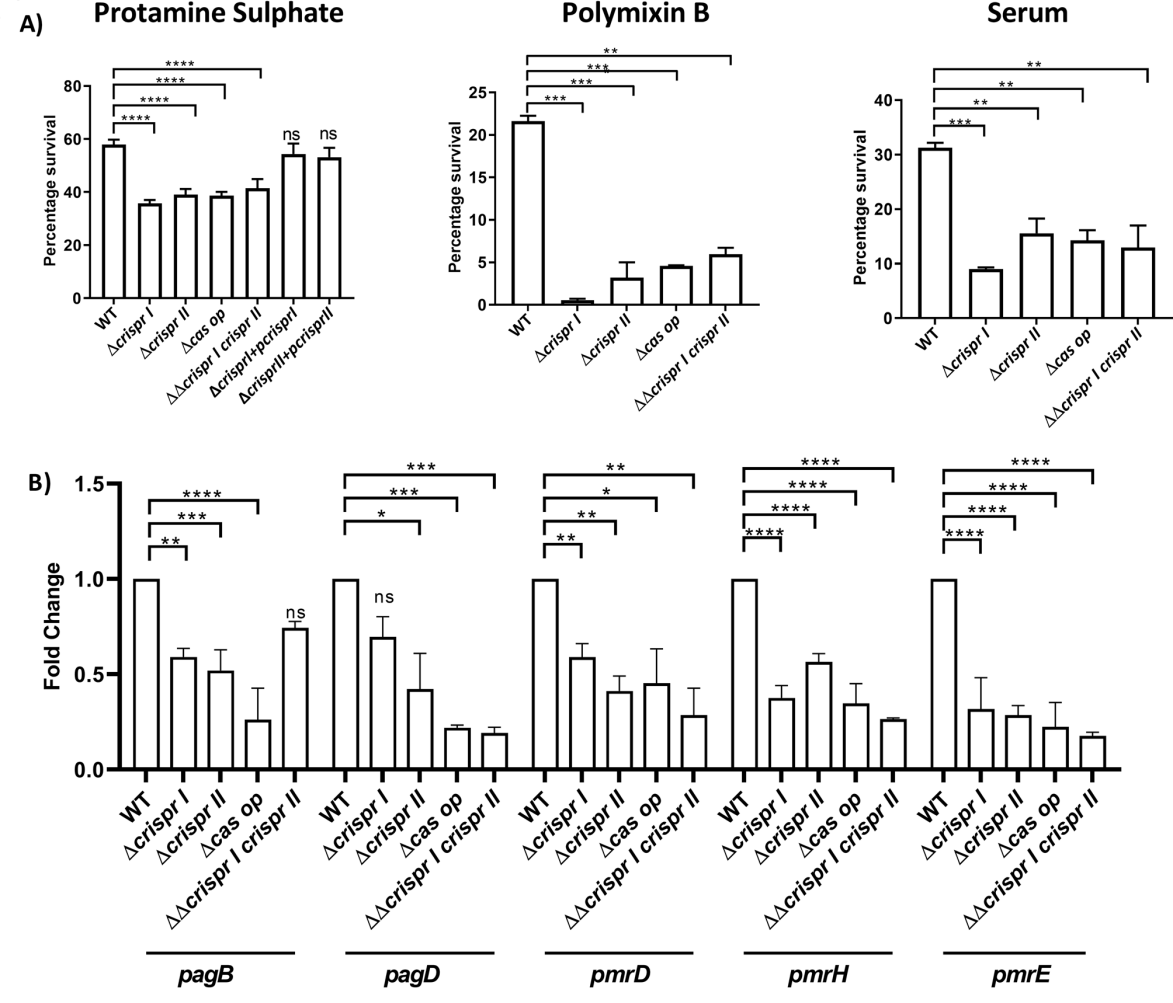
The knockout strains of CRISPR-Cas components show sensitivity towards antimicrobial peptides (AMP), and the complement system. **(A)** The strains were exposed to antimicrobials (i) AMPs- protamine sulfate (0.5 µg/mL), polymyxin B (0.5 µg/mL), and (ii) serum (20% FBS) for 1 h. For AMPs, the percentage survival was determined by analyzing CFU in both untreated and treated samples, the untreated samples were used as controls. Percentage survival in serum was determined by using heat-inactivated samples as control. **(B)** Total RNA isolated from late log-phase bacteria strains was used for cDNA synthesis, followed by qRT-PCR to assess the expression of polymyxin resistance (*pmr*) genes like *pagB*, *pagD*, *pmrH*, *pmrE*, *pmrD.* Relative expression of the gene was calculated using the 2 ^–ΔΔCt^ method, and normalized to reference gene *rpoD*. One-way ANOVA (Dunnett’s multiple comparison test) was used to determine significant differences between the WT and knockout strains, in at least three independent experiments, with at least 3 replicates in each. Error bars indicate SD. Statistical significance is shown as follows: *, *p* ≤ 0.05; **, *p* ≤ 0.01; ***, *p* ≤ 0.001; ****, *p* < 0.0001; and ns, not significant

It is known that the intestinal invasion by *Salmonella* evokes innate immune responses by the host. The intestinal epithelial cells reinforce the intestinal barrier function by releasing antimicrobial peptides (AMPs), while the immunity components in serum, like lysozyme and complement (also present in the intestine), restrict microbial colonization [21]. As the CRISPR-Cas knockout strains show attenuated virulence, we estimated their sensitivity against serum (complement system) and cationic AMPs like protamine sulfate and polymyxin B. Compared to the wildtype strain, the knockout strains showed ∼ 40–50% and ∼ 60–70% reduction in their percentage survival in the presence of protamine sulfate and polymyxin B, respectively (Fig. 3A). The knockout strains showed ∼ 15–30% reduction in survival in the presence of serum (Fig. 3A).

To withstand the harm caused by polymyxins, Gram-negative bacteria alter their lipopolysaccharides [22] with the help of two-component systems, like PhoP/PhoQ and PmrA/PmrB [23]. These systems trigger the upregulation of operons like *pmrCAB* and *arnBCADTEF-pmrE* (*pmrHFIJKLM-ugd*), facilitating the synthesis and transfer of PEtN and L-Ara4N to lipid A [23]. Therefore, we examined the expression patterns of several polymyxin resistance *(pmr*) genes, namely *pagB*, *pagD*, *pmrH*, *pmrE*, *pmrA*, and *pmrD.* The knockout strains exhibited a decrease in expression levels of *pagB* (1.4-to-2.5-fold), *pagD* (∼ 1.4 to 2.5 fold), *pmrD* (∼ 2 to 2.5 fold), *pmrH* (∼ 2.5 to 3 fold), and *pmrE* (2.5-fold) (Fig. 3B). However, there was no consistent change in the expression of the *pmrA* gene across all the strains (Supplementary Fig. S3).

Collectively, the results indicate that the knockout strains have an impaired ability to overcome innate immune barriers during the dissemination and intestinal infection phase.

### The CRISPR array and *cas* operon knockout strains show altered expression of *Salmonella* pathogenicity island (SPI-1 and SPI-2) genes

**Fig. 4:**
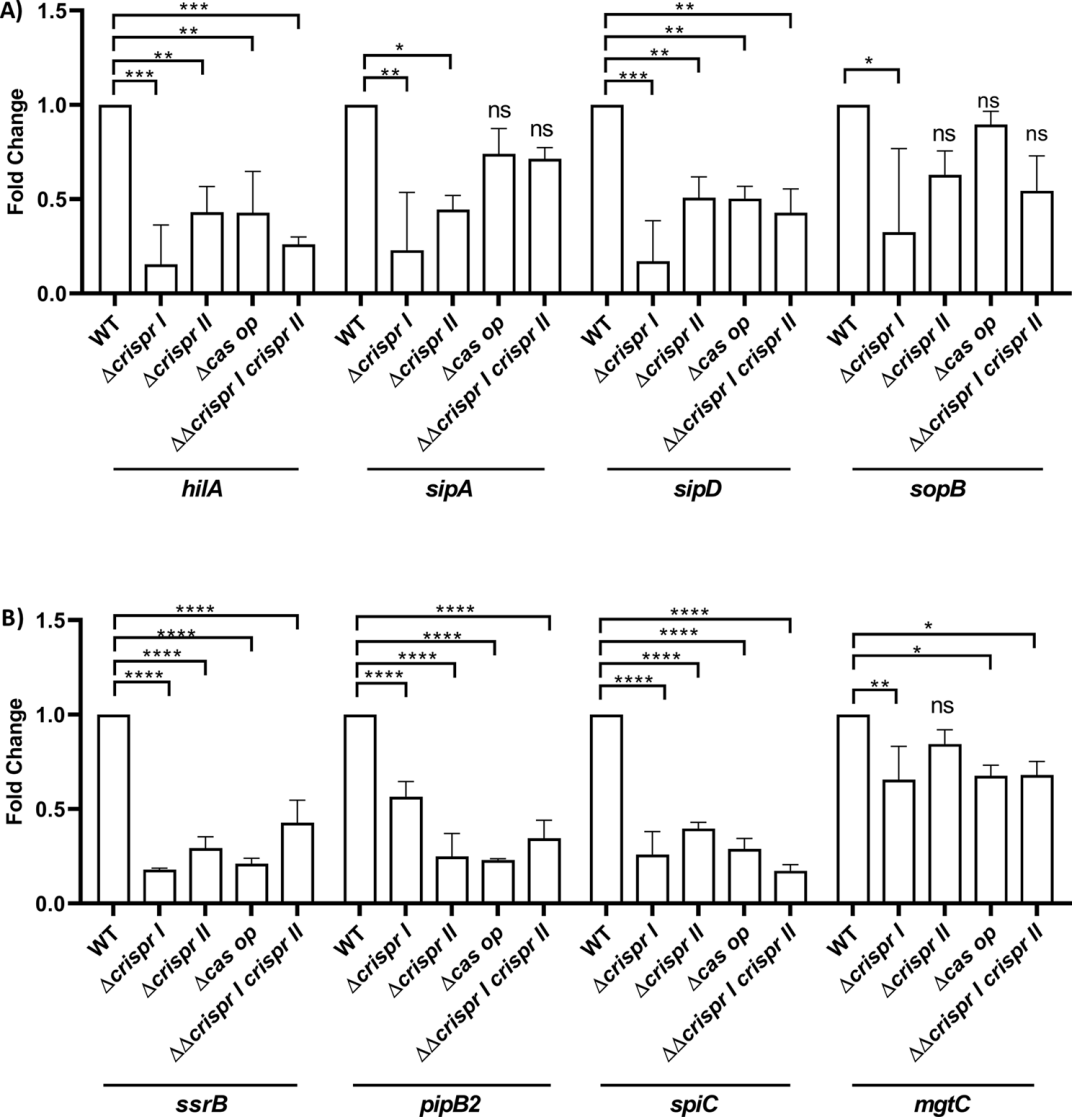
The CRISPR-Cas system regulates SPI-1, and SPI-2 genes expression. **(A)** The bacterial strains were cultivated in conditions (1:100 dilution in LB, followed by 8 h incubation) that promote SPI-1 activation. Subsequently, qRT-PCR was conducted on isolated RNA samples to assess the expression levels of key SPI-1 components, including transcriptional regulator-*hilA*, and SPI-1 effectors- *sipA*, *sipD*, *sopB*. **(B)** The bacterial strains were cultivated in SPI-2 inducing conditions (MgM- MES media) for 5 h, and qRT-PCR was performed from isolated RNA to check expression of SPI-2 effectors- *pipB2* and *spiC*, SPI-2 encoded transcriptional regulator-*ssrB*, and SPI-3 encoded protein-*mgtC*. Relative expression of the gene was calculated using the 2 ^–ΔΔCt^ method and normalized to the reference gene, *rpoD.* One-way ANOVA (Dunnett’s multiple comparison test) was used to determine significant differences between the WT and knockout strains, in at least three independent experiments, with at least 3 replicates in each. Error bars indicate SD. Statistical significance is shown as follows: *, *p* ≤ 0.05; **, *p* ≤ 0.01; ***, *p* ≤ 0.001; ****, *p* < 0.0001; and ns, not significant

To gain mechanistic insights into the regulation of pathogenesis by the CRISPR array and *cas* operon knockout strains, we checked the expression of effectors encoded by SPI-1 and SPI-2 pathogenicity island using RT-PCR. The SPI-1 is required during the intestinal phase of infection, delivering the effector proteins necessary for intestinal invasion and inflammation inside the host cells [24]. As the knockout strains were defective in the invasion, we first assessed the expression of SPI-1 regulatory genes like *hilA* and *h-ns*. All the knockout strains showed reduced expression (∼ 3–4 fold) of *hilA* (Fig. 4A). The *h-ns* gene did not show any difference in the expression among all the strains (Supplementary Fig. S4). Next, to envisage the impaired invasion ability of the knockout strains, we analyzed the expression of a few critical SPI-1 effectors, *sipA*,* sipD*, and *sopB*. The knockout strains showed ∼ 2-2.5 fold and ∼ 1.5-2 fold reduced expression of *sipA* and *sipD*, respectively. However, *sopB* was downregulated by only ∼ 1.4-fold in the knockout strains, except for ΔΔ*crisprI crisprII*, which showed more than two-fold downregulation (Fig. 4A). The secreted SPI-1 profile also reflects the downregulation of SPI-1 genes (Supplementary Fig. S8).

Following the epithelial cell invasion, *Salmonella* employs SPI-2-encoded effector proteins to form a permissive-replicative niche in *Salmonella* Containing Vacuole (SCV) [25]. SsrAB, a two-component system, regulates the expression of SPI-2 effector proteins like PipB2, SpiC, etc [26]. Thus, we checked the expression of representative SPI-2 genes and its regulator SsrAB in the strains grown in MgM-MES media for 4 h where the SPI-2 genes are expressed maximally [20]. The expression of the SPI-2 effector, *pipB2* and *spiC*, and the transcriptional regulator, *ssrB*, was downregulated by more than 2-fold in all the knockout strains (Fig. 4B). The low Mg^2+^ milieu of SCV promotes MgtC expression, a virulence protein required for intracellular replication inside macrophages [27]. Hence, we also evaluated the expression of *mgtC* in strains grown in MgM-MES media. All the knockout strains show 1.25-1.6-fold downregulation in *mgtC* expression (Fig. 4B).

### The CRISPR array and *cas* operon knockout strains show reduced survival against oxidative response but induce similar oxidative responses in macrophages as that of the wildtype

After the epithelial barrier is breached, *Salmonella* are engulfed by macrophages, where they encounter oxidative and nitrosative stress [28]. The diminished intracellular proliferation of the knockout strains in macrophages may be attributed to their ability to induce elevated production of free radicals, leading to enhanced cellular death. Alternative explanations for the reduced survival include the strains’ increased susceptibility to oxidative and nitrosative stress responses triggered by the host cells.

At first, we assessed the viability of the knockout strains in the presence of oxidative stressors like H_2_O_2_ and nitric oxide. In the presence of H_2_O_2_, the percentage survival of the knockout strains was significantly reduced by ∼ 60–70% for Δ*crisprI*, Δ*crisprII*, Δ*cas op*, and ΔΔ*crisprI crisprII* when compared to that of the wildtype (Supplementary Fig. S5A). However, all the strains exhibited similar sensitivity to sodium nitrite (Supplementary Fig. S6).

**Fig. 5:**
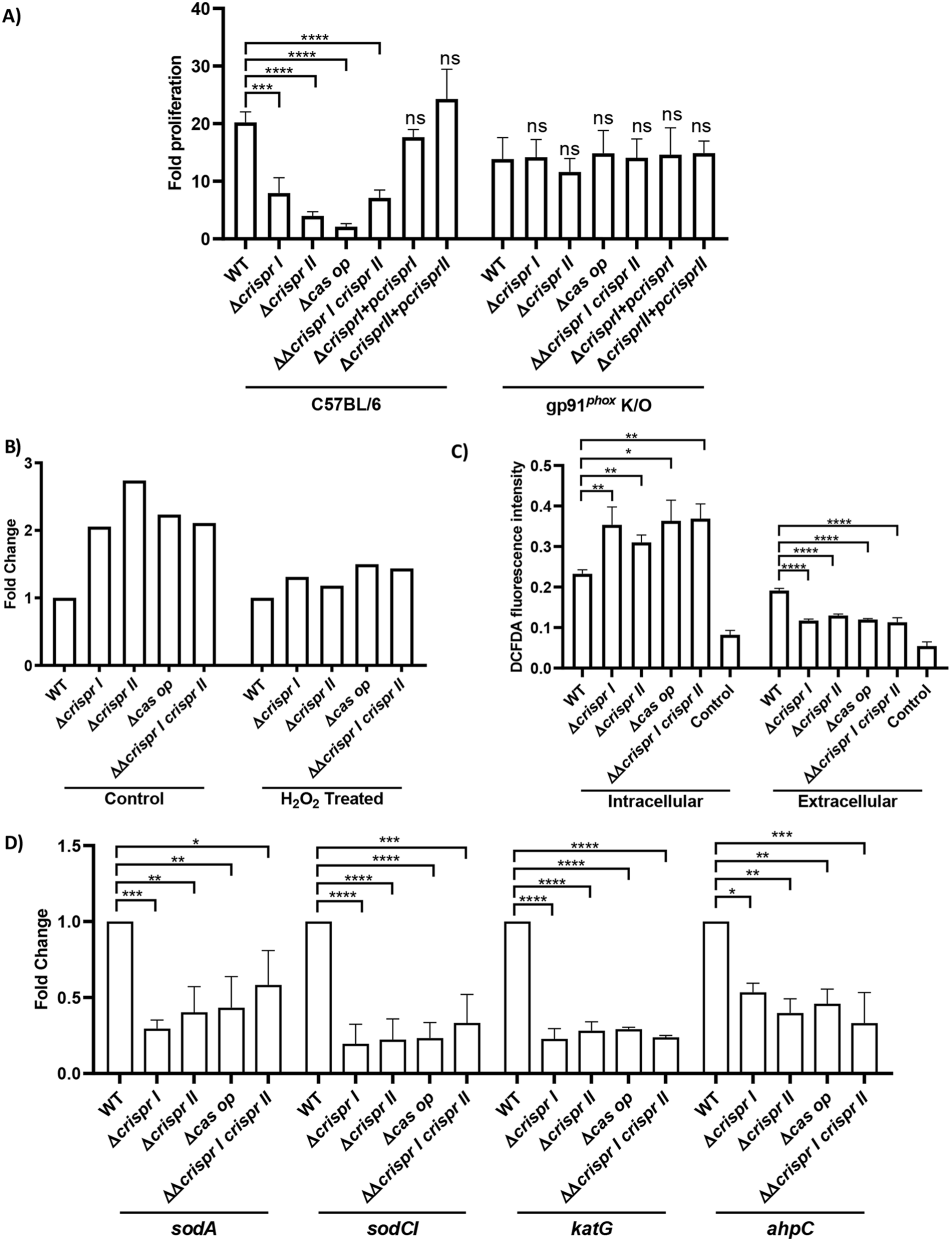
The knockout strains of CRISPR-Cas components are ROS-susceptible owing to elevated H_2_O_2_influx, and reduced antioxidant genes expression. **(A)**The peritoneal macrophages were infected with *S.* Typhimurium strain 14028s wildtype (WT), CRISPR (Δ*crisprI*, Δ*crisprII*, and ΔΔ*crisprI crisprII*) and *cas* operon * (*Δ*cas op*) knockout strains along with their respective complements (Δ*crisprI +* p*crisprI* and Δ*crisprII +* p*crisprII*). Intracellular fold proliferation was calculated by normalizing the CFU count of intracellular bacteria at 16 h to 2 h. **(B)** The bacterial strains were cultivated in MgM-MES media, with and without H_2_O_2_ for 8 h. RNA was isolated, followed by qRT-PCR analysis of *ompW*. Relative expression of the gene was calculated using the 2 ^-ΔΔCt^ method and normalized to reference gene *rpoD*. **(C)** The bacterial strains were cultivated in MgM-MES media until they reached an OD_600nm_ ∼ 0.5. They were then incubated in the dark for 5 min with 1 mM H_2_O_2_. The H_2_O_2_ levels in both extracellular and intracellular fractions were measured using H_2_DCFDA. The H_2_O_2_ untreated sample was used as a control. **(D)** The bacterial strains were cultivated in SPI-2 inducing conditions (MgM-MES media), and qRT-PCR was performed from isolated RNA to evaluate the expression of ROS detoxifying enzymes, superoxide dismutases (*sodCI* and *sodA*), catalase (*katG*), and peroxidase (*ahpC*). Relative expression of the gene was calculated using the 2 ^–ΔΔCt^ method and normalized to the reference gene, *rpoD.* One-way ANOVA (Dunnett’s multiple comparison test) was used to determine significant differences between the WT and knockout strains, in at least three independent experiments, with at least 3 replicates in each. Error bars indicate SD. Statistical significance is shown as follows: *, *p* ≤ 0.05; **, *p* ≤ 0.01; ***, *p* ≤ 0.001; ****, *p* < 0.0001; and ns, not significant

Following this, we assessed the strains’ intracellular survival in macrophages with enhanced (LPS induced) and suppressed (gp91^*phox*^ knockout) oxidative response. Similar to the data with the wildtype peritoneal macrophages, the CRISPR-Cas knockout strains were phagocytosed less by ∼ 50–60% by the gp91^*phox−/−*^ macrophages when compared to that of the wildtype *Salmonella* (Supplementary Fig. S5B). As anticipated, the intracellular proliferation of the CRISPR-Cas knockout strains was comparable to that of the wildtype (Fig. 5A). Next, we assessed the proliferation of these strains in macrophages with enhanced and suppressed ROS production (Supplementary Fig. S7). The knockout strains exhibited comparable intracellular proliferation to that of wildtype in suppressed oxidative response conditions (Supplementary Fig. S7C).

When assessed for induction of oxidative response in the macrophages by the knockout strains, we found comparable intracellular ROS levels in macrophages infected with the knockout or wildtype strains (Supplementary Fig. S7A). Likewise, we did not observe any significant difference in the extracellular nitric oxide produced by these infected cells (Supplementary Fig. S7B).

### CRISPR array and *cas* operon knockout strains exhibit susceptibility to ROS due to amplified H_2_O_2_ influx and diminished expression of antioxidant genes

Given the susceptibility of the knockout strains to ROS, we explored the underlying reasons for this. Outer membrane porin, OmpW, aids the influx of H_2_O_2_ in *Salmonella* [29], and the *ompW* null mutants of *E. coli* and *Salmonella* are resistant to oxidative stress [29, 30]. Additionally, the *ompW* null mutants show enhanced surface-attached biofilm in *Cronobacter sakazakii* [31, 32]. The CRISPR-Cas knockout strains showed reduced H_2_O_2_ tolerance (Supplementary Fig. S5A) and lesser surface-attached biofilm compared to that of wildtype [15]. We hypothesized that the upregulation of *ompW* in the knockout strains might lead to the observed phenotypes. With this antecedent, we assessed the expression of *ompW* in the knockout strains. The *ompW* expression was 2-fold higher for Δ*crisprI* and Δ*cas op*, while in Δ*crisprII* and ΔΔ*crisprI crisprII*, it was 3-fold higher than that of the wildtype (Fig. 5B). Consequently, the H_2_O_2_ uptake was high in the knockout strains (Fig. 5C). H_2_O_2_ is known to repress the expression of *ompW* in *S*. Typhimurium [29]. As expected, the H_2_O_2_ treatment reduced the expression of *ompW*, and the difference in the expression between the knockout strains and wildtype reduced to 1 to 1.3-fold wildtype (Fig. 5B). Considering that the *ompW* is expressed at similar levels in H_2_O_2_ primed wildtype and knockout strains, we next assessed the survival of the knockout strains post-priming. The percentage survival of the primed knockout strains was similar to that of the primed wildtype (Supplementary Fig. S8).

To combat the oxidative stress response generated by the host cell, *Salmonella* employs an array of antioxidant enzymes like superoxide dismutase, catalase, and peroxidase to detoxify ROS [33]. As the knockout strains are sensitive to H_2_O_2_, we analyzed the expression of antioxidant genes (*sodA*,* sodCI*,* katG*, and *ahpC)* in the strains grown in MgM-MES media [19]. The antioxidant genes were repressed in all the knockout strains (Fig. 5D), explaining their reduced survival in H_2_O_2_.

## Discussion

Our present study demonstrates that knocking out the CRISPR-Cas components attenuates the virulence of *S*. Typhimurium, reducing its invasion and proliferation in host cells. The results are similar to those observed for *S*. Enteritidis [9]. However, one should note that the *cas3* knockout strain used in this study has other *cas* genes upregulated, and the CRISPR array is intact [9].

**Fig. 6:**
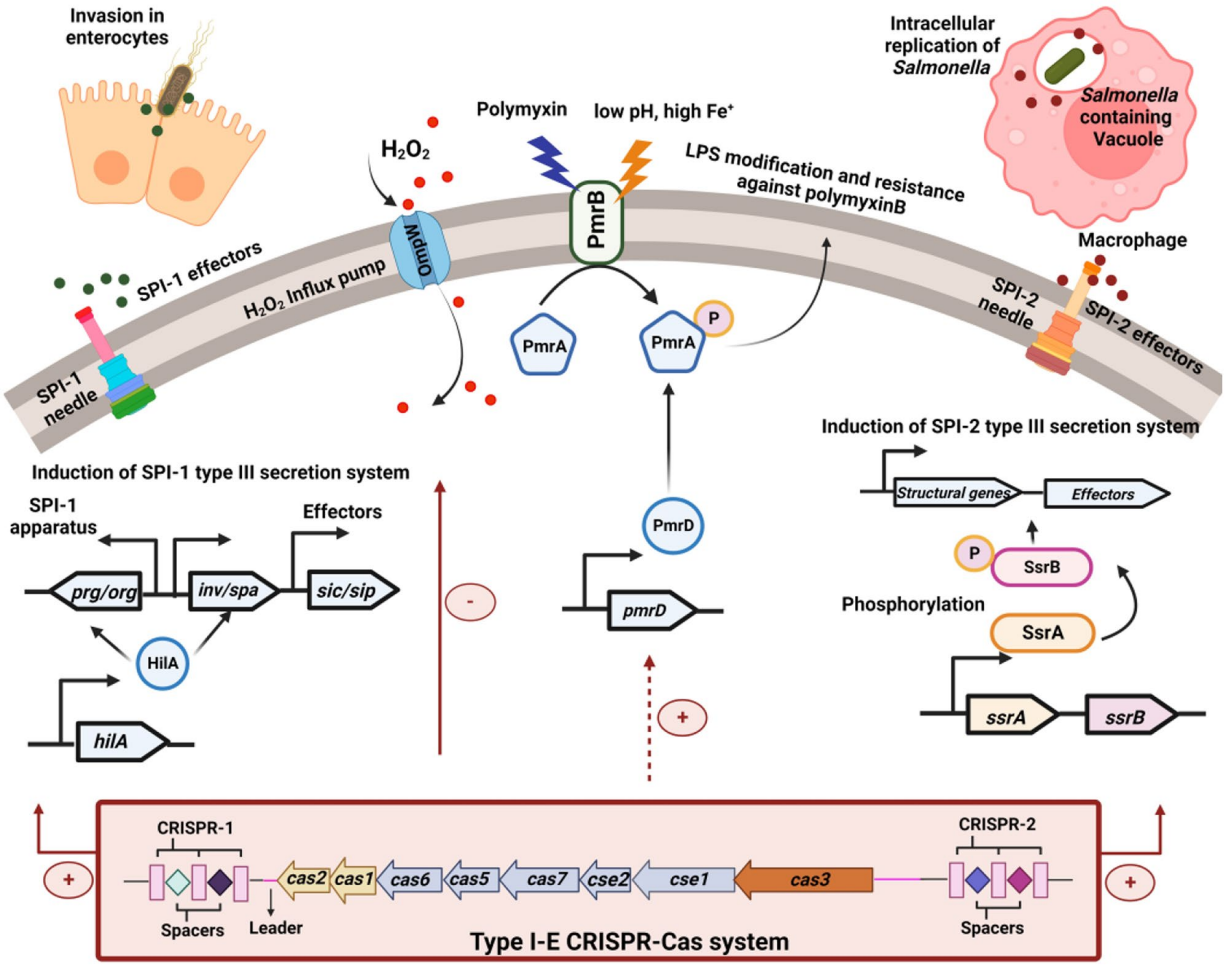
Deletion of the CRISPR-Cas system attenuates *Salmonella* pathogenicity. Proposed mechanisms of the type I-E CRISPR-Cas system in regulating *Salmonella* pathogenesis via modulation of SPI-1 and SPI-2 genes. We propose that the CRISPR-Cas system positively regulates *hilA* (direct regulation via complementary base-pairing between crRNA and gene) whereby it upregulates the expression of SPI-1 apparatus and effector proteins (direct regulation of *sipA*) involved in the invasion of enterocytes by *Salmonella*. The intestinal epithelial cells reinforce the intestinal barrier function by releasing antimicrobial peptides. The CRISPR-Cas system appears to indirectly regulate (red dotted lines) *pmr* genes to provide resistance against antimicrobial peptides (AMPs). Within the *Salmonella* containing vacuole (SCV) of macrophages, the bacteria shut down its SPI-1 system and activates the SPI-2-encoded SsrAB system in response to the acidic milieu. The SsrAB system further activates the SPI-2 encoded genes. The CRISPR-Cas may be positively regulating SsrB (direct regulation) to trigger activation of SPI-2 encoded structural genes and effector proteins (direct regulation of *pipB2*) to aid intracellular proliferation and survival of *Salmonella*. In addition, the CRISRP-Cas system negatively regulates OmpW (direct regulation) during oxidative stress, thereby aiding in *Salmonella’*s survival. Taken together, the CRISPR-Cas system positively regulates *Salmonella* pathogenesis. The figure was created using Biorender

During the initial phase of infection, *Salmonella* invades the intestinal epithelial cells using SPI-1-encoded effectors (Fig. 6) [34]. The expression of these effectors is regulated by *hilD* and *hilA* [35]. SPI-1 translocases SipB, SipC, and SipD are essential for the attachment of bacteria to the target cells [36], and SipA is required for the efficient invasion. Following membrane ruffling [37], *Salmonella* outer membrane proteins (Sops) control cytoskeletal rearrangement during the invasion and regulate polymorphonuclear leukocyte influx [38]. In-vivo experiments demonstrate that SopB is required during the initial invasion process and in the later stage of murine salmonellosis [39]. The knockout strains show decreased expression of the *hilA*,* sipA*, *sipD*, and *sopB*, explaining their decreased invasion phenotype in in-vitro and in-vivo models (Fig. 6). Partial complementarity between CRISPR spacers and *sipA* and *sopB* suggests the plausible involvement of the CRISPR-Cas system in modulating the regulation of these specific genes (Supplementary Fig 10). Furthermore, modifications in the LPS O-antigen structure have been shown to significantly influence the uptake of *S*. Typhimurium by macrophages, primarily by affecting the translocation of SipA during host cell invasion [40, 43]. The CRISPR-Cas knockout strains show altered LPS structure [15], which may also contribute to the reduced expression of *sipA*.

Moreover, adhesins like flagella and Curli inherently contribute to adhesion and invasion into the epithelial cells [41, 42]. Our previous studies demonstrated reduced expression of the flagellar and curli genes in the CRISPR-Cas knockout strains [15], thereby explaining their attenuated invasion in epithelial cells. *Salmonella* relies on the flagellar subunit FljB to invade the epithelial cells and cross the mucosal barrier produced by the goblet cells [21]. The reduced invasion of the CRISPR-Cas knockout strains in differentiated HT-29 cells that produce mucin over undifferentiated cells could be explained through the reduced *fljB* expression in these strains [15]. This, along with the increased sensitivity of the knockout strains to serum complement and AMPs in the intestinal lumen, could explain their reduced colonization of the PP. It is reported that LPS modification affects bacterial susceptibility to complement [43], AMPs [44], and phagocytosis [40, 43]. Reportedly, the LPS modifying genes, along with *rfa* (coding for LPS core synthesis) and *rfb* genes (O-antigen synthesis), are repressed in the CRISPR-Cas knockout strains [15], thereby explaining their reduced phagocytosis and increased sensitivity to AMPs and complement.

Our data demonstrate altered expression of different pathogen-associated molecular patterns in the CRISPR-Cas knockout strains. Yet, they fail to show any differences in the induction of oxidative and nitrosative response in RAW 264.7 cells compared to that induced by the wild type strain. Additionally, the knockout strains survive and grow better in MgM-MES media (Supplementary Fig S9). Despite this, the knockout strains have attenuated intracellular proliferation, possibly due to their increased susceptibility to H_2_O_2_*via* the increased expression of OmpW, an importer of H_2_O_2_ (Fig. 6) [29]. This aligns with previous studies reporting that the CRISPR-Cas system can regulate outer membrane proteins in *S*. Typhi [45]. As the Omp protein is widely distributed in the Enterobacteriaceae family [46], the question is, can the CRISPR-Cas system regulate *omp* expression in other members of the Enterobacteriaceae family? This question needs further exploration. OMPs and LPS help the bacteria tolerate different environmental stresses, including H_2_O_2_. *Salmonella* O-antigen capsule mutants are susceptible to H_2_O_2_ under biofilm conditions [47]. Thus, the altered LPS profile of the knockout strains [15] could also contribute to their H_2_O_2_ sensitivity. The sensitivity of the knockout strains to H_2_O_2_ corroborates the findings from our previous study, where a few cells in the CRISPR-Cas knockout strains become filamentous at 24 h [15]. This indicates a potential induction of reactive oxygen species (ROS) during biofilm formation, and a few cells become filamentous in response to oxidative stress [15]. As the biofilm formation progressed, the nutrient deprivation could have accelerated ROS that could have been influxed in the knockout strains, resulting in reduced viability of the strains at 24 h.

Apart from intracellular ROS, *Salmonella* also encounters extracellular H_2_O_2_ during the intestinal phase of infection [48], employing an array of oxidative enzymes to scavenge and degrade H_2_O_2_ molecules. Such enzymes include the cytoplasmic catalases (*katE*,* katG*, and *katN*), peroxidases (*ahpC*, *tpx*, and *tsaA*), superoxide dismutases (*sodA* and *sodB*), and the periplasmic superoxide dismutases (*sodCI*) [33]. The CRISPR-Cas knockout strains showed downregulation of these enzymes (one representative of each group), thereby displaying increased sensitivity against H_2_O_2_ and reduced survival within the macrophages and mice.

Besides the mechanisms mentioned above, the coordinated action of other virulence determinants plays a major role in governing the survival and replication of *Salmonella* within SCV. Among them, MgtC is one such virulence factor that promotes intra-phagosomal replication under low Mg^2+^ conditions [27]. It also promotes *Salmonella* virulence by negatively regulating cellulose production [49]. Deletion of *mgtC* attenuates *Salmonella* virulence in the mammalian host [49]. Interestingly, our study displayed such a relation wherein all the CRISPR-Cas knockout strains showed decreased *mgtC* expression and enhanced cellulose secretion [15]. Thus supporting their impaired intracellular survival in phagocytic cells and in-vivo models.

During in-vivo infection, *Salmonella* is transported from the intestinal lumen and PP to the MLN, liver, and spleen as extracellular bacteria or within the phagocytic cells [50]. Thus, the innate immune barrier, like resistance against AMPs, ROS, and serum proteins, must be overcome to disperse systemically. The CRISPR-Cas knockout strains show reduced survival in the presence of AMPs, H_2_O_2,_ and serum, explaining their attenuated colonization and systemic spread. The differential expression of *pmr* genes (Fig. 6) and the altered LPS profile in the knockout strains could be one of the reasons for their increased sensitivity against AMPs and serum. However, the role of the CRISPR-Cas system in regulating other factors like OMPs, Rck, and siderophore cannot be ruled out and needs further exploration. The acidic milieu inside the immune cells activates the SsrAB system, which in turn activates the expression of SPI-2 encoded effector proteins like SpiC, PipB2, etc. (Fig. 6). Decreased expression of the SPI-2 effectors and other virulence genes like *mgtC*,* katG*,* sod* and *ahpC* in the knockout strains could explain their sensitivity to antimicrobial defenses, like ROS [51]. This could have attenuated their virulence, impacting the colonization of *C. elegans* and mice.

Our study suggests that the CRISPR-Cas system regulates the virulence genes of *S*. Typhimurium. To explain this regulation, we hypothesize that the crRNA-Cascade complex may bind to the gene due to a partial spacer match with the gene, thereby interfering with the transcription of these genes. Studies elsewhere suggest that five bp complementarity between the crRNA and target is sufficient for the type I-E Cascade complex to bind the target [52]. Through in-silico analysis, we found partial complementarity between CRISPR spacers and key virulence regulators (*ssrB*, *pipB2*)/other virulence-related genes (*sodA*, *katG*, *ompW*) (Supplementary Fig S10), suggesting potential regulatory interactions. Apart from the genes tested in our study, the system could be modulating the other regulators like *phoP*,* omp*, etc., which may indirectly impact the expression of the genes studied but need further validation.

Our findings indicate that the CRISPR-Cas system may influence the expression of virulence factors like antioxidant genes (*sodA*,* sodCI*,* katG*, and *ahpC*), SPI-1 and SPI-2 effectors, thereby playing a role in *Salmonella’s* virulence during host infection (Fig. 6). We observed that ∆*crisprIcrisprII* shows lesser inflammatory response (IFN-γ induction) than the ∆*crisprI* and ∆*crisprII*. There is a possibility that some of the PAMPs that we have not tested are regulated differently, but it is outside the scope of this study, and we cannot justify the observation with current data and understanding.

Further investigation is needed to unveil the specific regulatory mechanisms the CRISPR-Cas system exhibits, such as the binding kinetics of the crRNA-Cascade to the target genes.

## Electronic supplementary material

Below is the link to the electronic supplementary material.

Supplementary Material 1

Supplementary Material 2

## Data Availability

No datasets were generated or analysed during the current study.
