# Supplementary material for: CRISPR-Cas system positively regulates virulence of Salmonella enterica serovar Typhimurium
Source: Gut Pathog. 2024 Oct 26;16:63. doi: 10.1186/s13099-024-00653-5 (PMC11514906; doi:10.1186/s13099-024-00653-5)
Supplement: Supplementary file 2 — Supplementary Material 2 [file 13099_2024_653_MOESM2_ESM.docx]

**Supplementary Figure S1: CRISPR-Cas knockout strains are attenuated in intracellular survival.** HT-29 (non-polarized),and RAW 264.7 macrophage cell lines polarized, were infected with *S.* Typhimurium strain 14028s wildtype (WT), CRISPR (Δ*crisprI,* Δ*crisprII,* and ΔΔ*crisprI crisprII*) and *cas operon (*Δ*cas op*) knockout strains along with their respective complements (Δ*crisprI+*p*crisprI* and Δ*crisprII+*p*crisprII*). **(A)** The percentage of invasion/phagocytosis in intestinal epithelial cells was calculated using CFU analysis of the infected cell lysate and the pre-inocula used for infection. **(B)** Fold proliferation was calculated by normalizing the CFU at 16 h to 2h. One-way ANOVA (Dunnett’s multiple comparison test) was used to determine significant differences between the WT and knockout strains, in at least three independent experiments, with at least 3 replicates in each. Error bars indicate SD. Statistical significance is shown as follows: *, p ≤ 0.05; **, p ≤ 0.01; ***, p ≤0.001; ****, p < 0.0001; and ns, not significant.


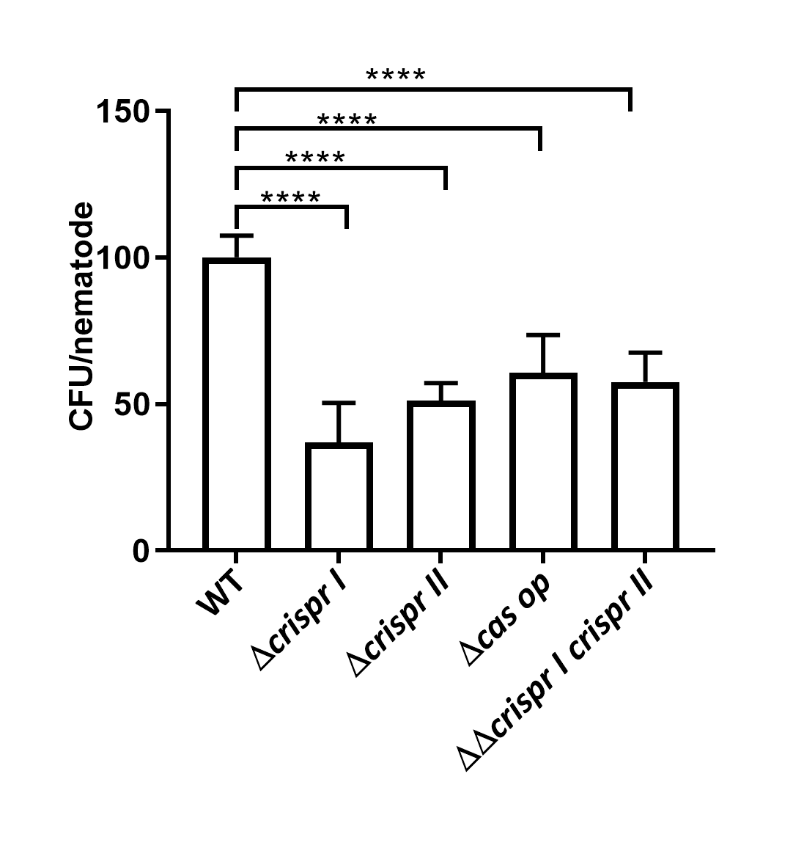


**Supplementary Figure S2: The CRISPR-Cas knockout strains show impaired colonisation in *in-vivo* model organism, *C. elegans.*** L4 synchronized worms were fed on mCherry tagged strains of *S.* Typhimurium strain 14028s wildtype (WT), CRISPR (Δ*crisprI,* Δ*crisprII,* and ΔΔ*crisprI crisprII*), *cas operon (*Δ*cas op*) knockout strains, and *E .coli*-OP50 for 12 h. 24 h post-infection, worms were crushed to estimate the CFU/nematode. One-way ANOVA (Dunnett’s multiple comparison test) was used to determine significant differences between the WT and knockout strains, in at least three independent experiments, with at least 3 replicates in each. Error bars indicate SD. Statistical significance is shown as follows: *, p ≤ 0.05; **, p ≤ 0.01; ***, p ≤0.001; ****, p < 0.0001; and ns, not significant.


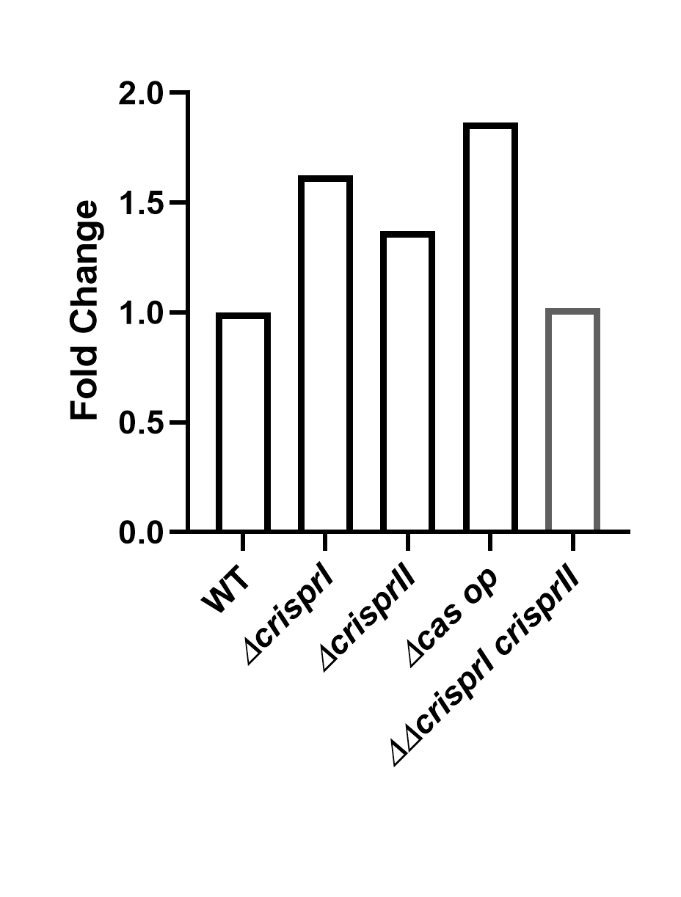


**Supplementary Figure S3: The *pmrA* expression lacks a consistent pattern in CRISPR-Cas knockout strains.** Total RNA isolated from late log-phase bacteria strains was used for cDNA synthesis, followed by qRT-PCR to assess the expression of *pmrA.* Relative expression of the gene was calculated using the 2 ^–ΔΔCt^  method, and normalized to reference gene *rpoD*.


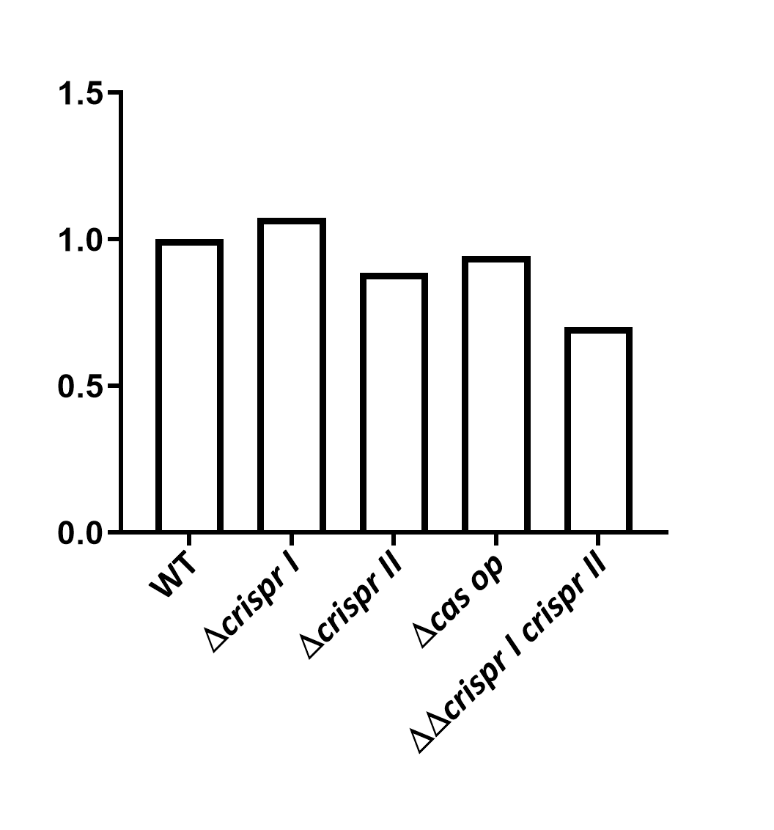


**Supplementary Figure S4: CRISPR-Cas knockout strains showed no differences in the expressions of global repressor, *h-ns*.** Total RNA isolated from late log-phase bacteria strains was used for cDNA synthesis, followed by qRT-PCR to assess the expression of *h-ns.* Relative expression of the gene was calculated using the 2 ^–ΔΔCt^ method, and normalized to reference gene *rpoD*.

**Supplementary Figure S5: CRISPR-Cas knockout strains’ diminished proliferation in phagocytic cells could be linked to their susceptibility to ROS. (A)** Overnight-grown strains were exposed to 1 mM H_2_O_2_ for 2 hours at 37°C in the dark. CFU counts were then determined, and percentage survival was calculated by normalizing treated samples to untreated controls. **(B)** The percentage of phagocytosis by peritoneal macrophages derived from GP91*^phox^* knockout mice was determined through CFU analysis of infected cell lysates. One-way ANOVA (Dunnett’s multiple comparison test) was used to determine significant differences between the WT and knockout strains, in at least three independent experiments, with at least 3 replicates in each. Error bars indicate SD. Statistical significance is shown as follows: *, p ≤ 0.05; **, p ≤ 0.01; ***, p ≤0.001; ****, p < 0.0001; and ns, not significant.


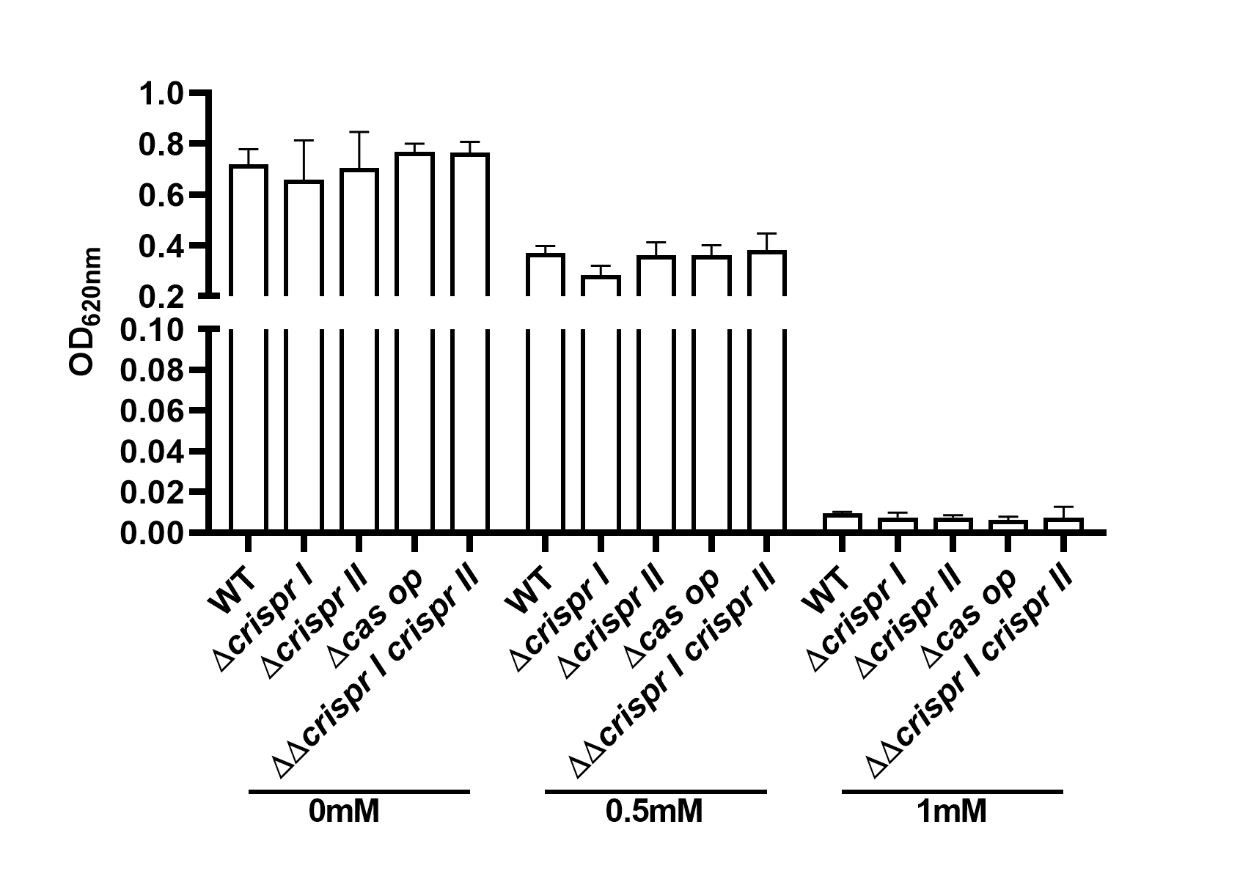


**Supplementary Figure S6: All strains are sodium nitrite-sensitive, with diminished growth at higher concentrations (1mM),** **while the growth rates of wild-type and knockout strains show no significant difference.** The bacterial strains were cultivated in MgM-MES media, with increasing concentration (0-1mM) of sodium nitrite, for 8 h, followed by the measurement of absorbance at 620nm.

**Supplementary Figure S7: CRISPR-Cas knockout strains’ diminished proliferation in phagocytic cells could be linked to their susceptibility to ROS.** RAW 264.7 macrophage cell lines were cultured both with and without LPS induction for 24 h, and subsequently were infected with the bacterial strains. N-acetylcysteine (NAC) was introduced to a specific set of LPS induced cell line to mitigate the effects of ROS. **(A)** The intracellular ROS production was estimated in infected cells 6 h post-infection using H_2_DCFDA. H_2_O_2_-treated, and uninfected cells were used as controls. **(B)** 16 h post-infection, the extracellular RNS production was estimated using a Griess reagent. NaNO_2_-treated, and uninfected cells supernatant were used as controls. **(C)** Intracellular fold proliferation in LPS induced and non-induced RAW 264.7 macrophage cell lines was calculated by normalizing the CFU count at 16 h to 2h. One-way ANOVA (Dunnett’s multiple comparison test) was used to determine significant differences between the WT and knockout strains, in at least three independent experiments, with at least 3 replicates in each. Error bars indicate SD. Statistical significance is shown as follows: *, p ≤ 0.05; **, p ≤ 0.01; ***, p ≤0.001; ****, p < 0.0001; and ns, not significant.


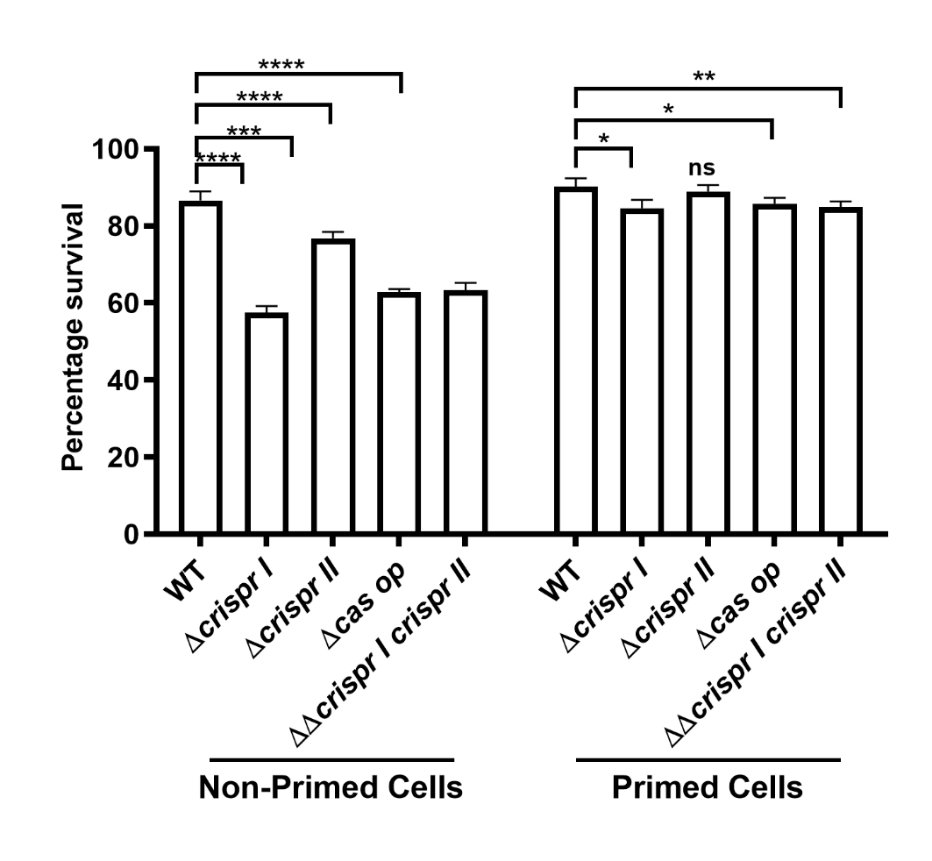


**Supplementary Figure S8: Pre-treatment with H_2_O_2_ improves survival of the CRISPR-Cas knockout strains.** The *S*. Typhimurium strain 14028s wildtype (WT), CRISPR (Δ*crisprI*, Δ*crisprII* and ΔΔ*crisprI crisprII*), and cas operon (Δ*cas op*) knockout strains were grown to OD600nm~0.4 in MH media. The bacterial cells were primed with 0.1 mM H_2_O_2_ for 30 min. The primed bacterial cells were exposed to 0 mM and 1 mM H_2_O_2_ for 8 h at 37°C in dark. Percentage survival was calculated by normalizing the treated samples to those of the untreated controls. One-way ANOVA (Dunnett’s multiple comparison test) was used to determine significant differences between the WT and knockout strains, in at least three independent experiments, with at least 3 replicates in each. Error bars indicate SD. Statistical significance is shown as follows: *, p ≤ 0.05; **, p ≤ 0.01; ***, p ≤0.001; ****, p < 0.0001; and ns, not significant.


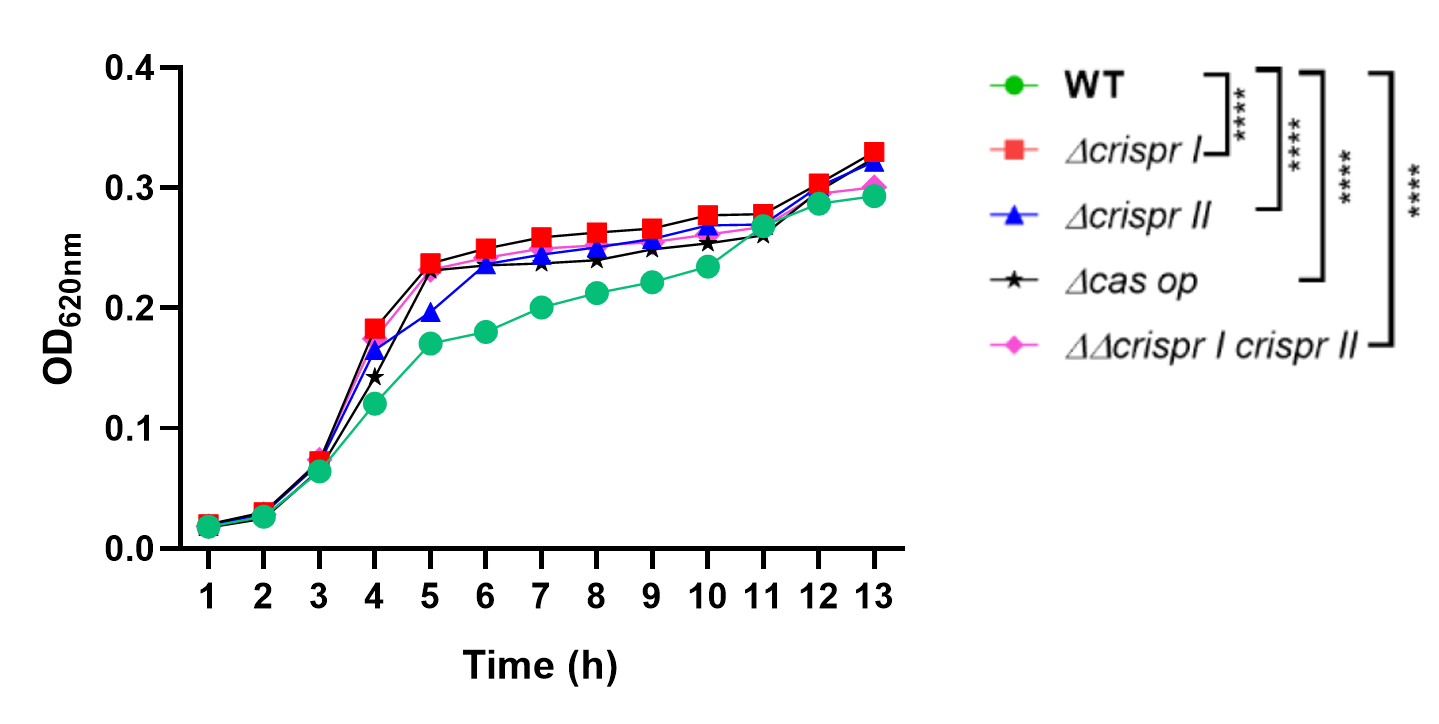


**Supplementary Figure S9: Growth Kinetics of the knockout strains of CRISPR-Cas components in F-media at pH-5.4.** The *S.* Typhimurium strain 14028s wildtype (WT), CRISPR (*ΔcrisprI, ΔcrisprII,* and *ΔΔcrisprI crisprII*) and *cas operon (Δcas op*) knockout strains were grown overnight in Luria broth, the bacterial cultures were sub-cultured in a ratio of 1:100 in MgM-MES media, pH 5.4, and incubated at 37°C under shaking conditions. The OD_620nm_ was monitored every hour and the graph was plotted


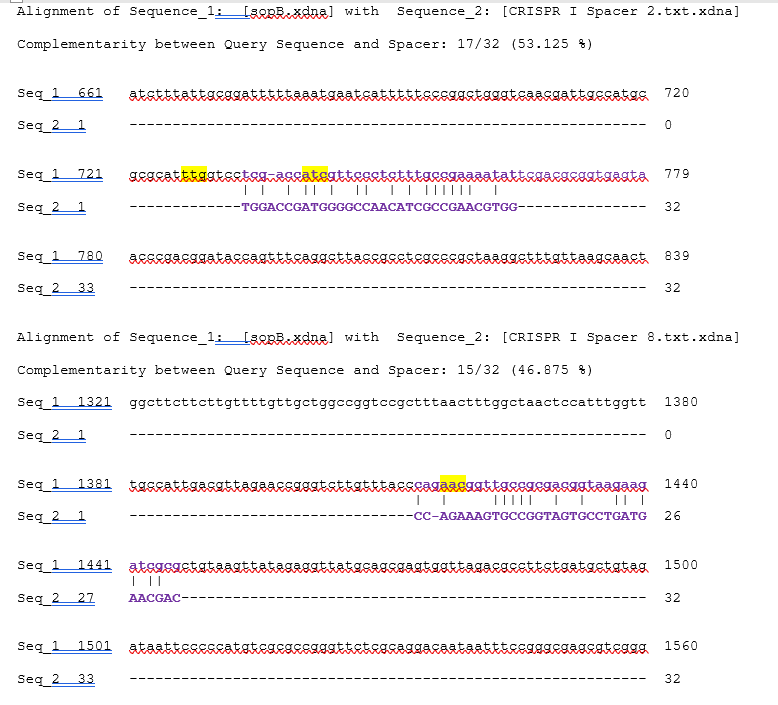


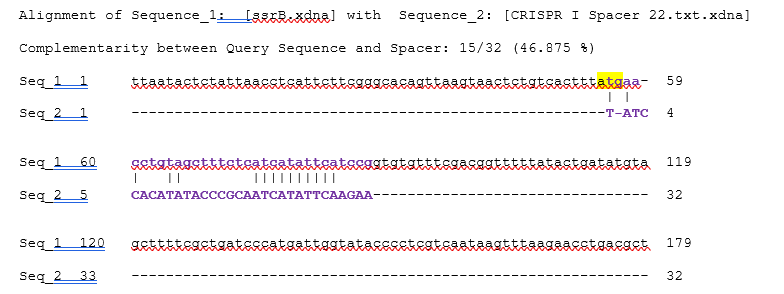


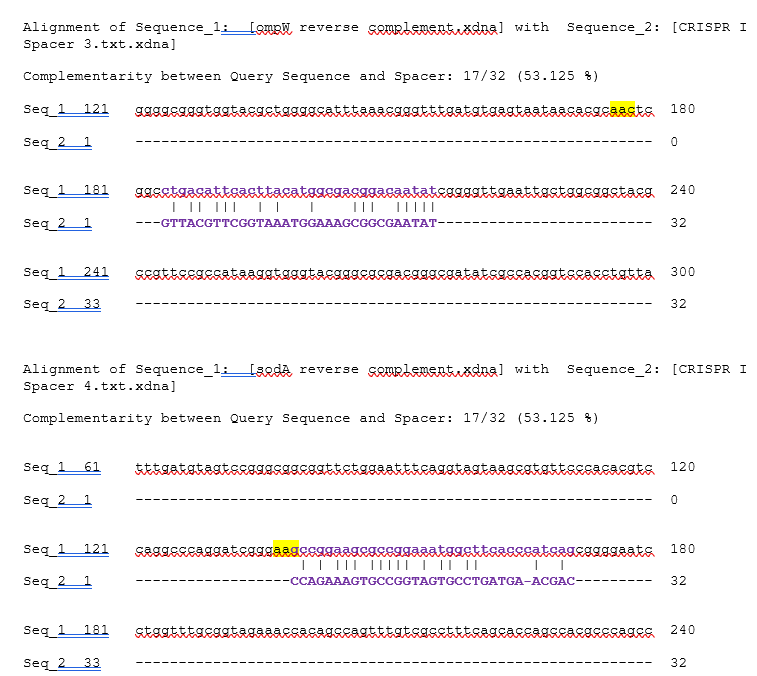


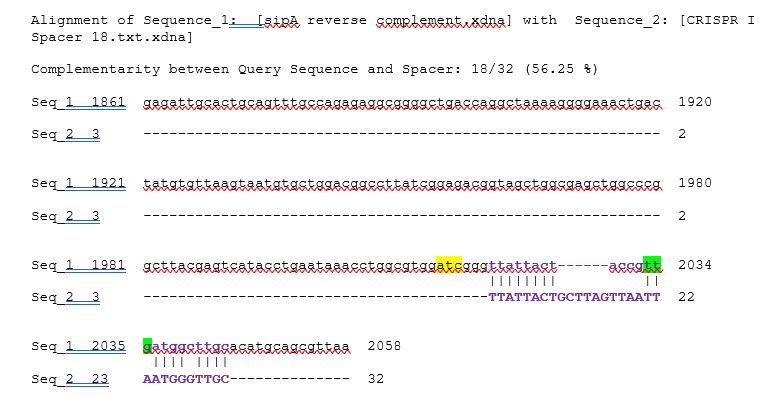


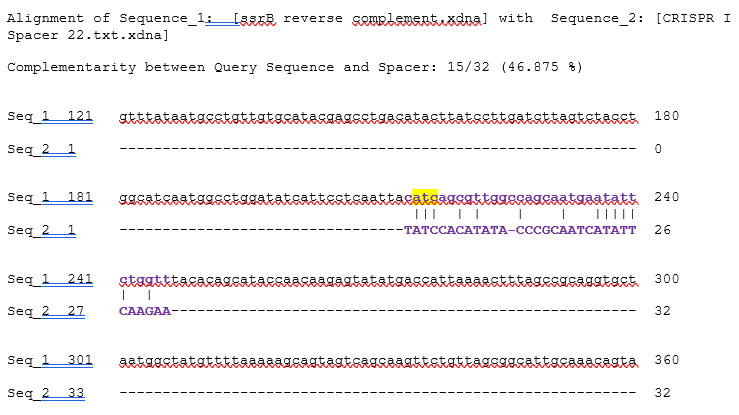


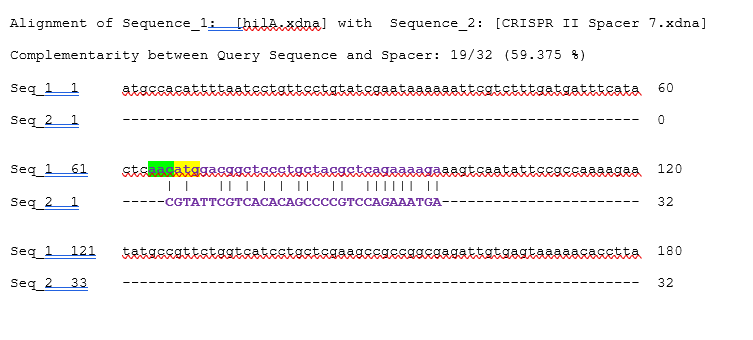


**Supplementary Figure S10: Partial complementarity between spacers in CRISPRI & CRISPRII array and various genes involved in *Salmonella* pathogenecity.** The coding and the reverse complement (template) sequence of the genes were extracted from a complete-genome sequence of Typhimurium str. 14028S, NCBI (GenBank: CP001363.1). The spacer sequences of CRISPRI and CRISPRII arrays were then aligned with coding and reverse complement of the genes using serial cloner version 2.6 software. The putative PAM sequences are highlighted.
